# 5-HT4 receptor agonists treatment reduces tau pathology and behavioral deficit in the PS19 mouse model of tauopathy

**DOI:** 10.3389/fncel.2024.1338502

**Published:** 2024-04-04

**Authors:** Shan Jiang, Eric J. Sydney, Avery M. Runyan, Rossana Serpe, Malavika Srikanth, Helen Y. Figueroa, Mu Yang, Natura Myeku

**Affiliations:** ^1^Taub Institute for Research on Alzheimer’s Disease and the Aging Brain, Columbia University Irving Medical Center, New York, NY, United States; ^2^Department of Pathology and Cell Biology, Columbia University Irving Medical Center, New York, NY, United States; ^3^The Institute for Genomic Medicine and Psychiatry, Columbia University Irving Medical Center, New York, NY, United States

**Keywords:** Alzheimer’s disease, ubiquitin proteasome system (UPS), tauopathy, 5-HT4 agonist, synaptic therapy

## Abstract

**Background:**

Accumulation of tau in synapses in the early stages of Alzheimer’s disease (AD) has been shown to cause synaptic damage, synaptic loss, and the spread of tau pathology through trans-synaptically connected neurons. Moreover, synaptic loss correlates with a decline in cognitive function, providing an opportunity to investigate therapeutic strategies to target synapses and synaptic tau to rescue or prevent cognitive decline in AD. One of the promising synaptic targets is the 5-HT4 serotonergic receptor present postsynaptically in the brain structures involved in the memory processes. 5-HT4R stimulation exerts synaptogenic and pro-cognitive effects involving synapse-to-nucleus signaling essential for synaptic plasticity. However, it is not known whether 5-HT4R activation has a therapeutic effect on tau pathology.

**Methods:**

The goal of this study was to investigate the impact of chronic stimulation of 5-HT4R by two agonists, prucalopride and RS-67333, in PS19 mice, a model of tauopathy. We utilized gradient assays to isolate pre- and post-synaptic compartments, followed by biochemical analyses for tau species and ubiquitinated proteins in the synaptic compartments and total brain tissue. Next, we performed kinetic assays to test the proteasome’s hydrolysis capacity in treatment conditions. Moreover, behavioral tests such as the open field and non-maternal nest-building tests were used to evaluate anxiety-like behaviors and hippocampal-related cognitive functioning in the treatment paradigm.

**Results:**

Our results show that 5-HT4R agonism reduced tauopathy, reduced synaptic tau, increased proteasome activity, and improved cognitive functioning in PS19 mice. Our data suggest that enhanced proteasome activity by synaptic mediated signaling leads to the enhanced turnover of tau initially within synapses where the receptors are localized, and over time, the treatment attenuated the accumulation of tau aggregation and improved cognitive functioning of the PS19 mice.

**Conclusion:**

Therefore, stimulation of 5-HT4R offers a promising therapy to rescue synapses from the accumulation of toxic synaptic tau, evident in the early stages of AD.

## Introduction

Besides main features such as the deposition of amyloid beta (Aβ) plaques and neurofibrillary tangles (NFT), other common characteristics of Alzheimer’s disease (AD) are synaptic dysfunction, structural damage on the synapses, and synaptic loss (Peng et al., 2022). The synaptic changes occur in the early stages of the disease before the symptoms of memory dysfunction (Chen et al., 2018), with recent studies suggesting that synaptic tau accumulation promotes synaptic impairment that contributes to the cognitive deficit (Xia et al., 2017). Tau is predominantly an axonal protein, and its presence in the synaptic compartments is low in physiological conditions. However, early in AD pathogenesis, tau is mislocalized to synapses with high levels of hyperphosphorylated and high molecular weight (HMW) tau in the post-synaptic compartments (DeVos et al., 2018; Schaler et al., 2021). Consequently, tau pathology spreads through synaptically connected neurons following the progression of the disease as defined by Braak stages I–VI (Braak and Braak, 1991) and demonstrated by PET imaging studies in patients with AD (La Joie et al., 2020) and progressive supranuclear palsy (PSP) (Cope et al., 2018). Notably, symptoms of memory dysfunction and later cognitive impairment correlate with the propagation of tau pathology from the hippocampus to the cortex (Qian et al., 2017).

Interestingly, the accumulation of synaptic hyperphosphorylated and HMW tau correlates with the accumulation of synaptic ubiquitinated proteins in AD brains and mouse models (Tai et al., 2012; Schaler et al., 2021), suggesting a disruption of synaptic proteasome-mediated proteolysis, which can further lead to synaptic damage and synaptic loss. Indeed, recent studies have reported that inhibition of synaptic protein degradation machinery can lead to the accumulation of tau in dendrites and the loss of dendritic spines (Balaji et al., 2018). Furthermore, mechanistic studies have shown that oligomers and aggregates can reduce proteasome activity by impairing the function of ATPase subunits of the 26S proteasome, presumably by obstructing the gate opening of the 26S proteasome (Myeku et al., 2016) or acting as allosteric inhibitors and stabilizing the closed gate conformation of the proteasome (Thibaudeau et al., 2018).

The recent understanding that tau propagation across neurons involves synaptic secretion to the extracellular space and subsequent internalization by the neighboring neurons has led to the development of immunotherapy clinical trials against extracellular tau species. However, the recent failure of anti-tau antibody therapies may hint that clearance of extracellular tau pool may not be an effective strategy as most pathological tau is intracellular (Congdon et al., 2022). Thus, increasing the turnover of intracellular tau, especially enhancing proteasome activity, which is recognized as the cell’s first defense mechanism against accumulating proteotoxicity, has been suggested as a new or additional approach to delay the onset or lessen the pathology in proteinopathy disorders (Myeku and Duff, 2018). Enhancing proteasome activity could have therapeutic potential but is still a relatively unexplored field. To this end, our team (Myeku et al., 2016) has recently shown that hyperphosphorylation of the 26S proteasome by PKA can mediate enhanced proteasome activity, reduced tauopathy, and cognitive decline in a mouse model. Subsequently, our group has shown that enhancing degradation of toxic tau restricted to synapses via stimulation of PAC1R, a Gs-coupled GPCR present on the membrane of post-synaptic compartment reduced the spread of tau, extracellular tau seeds, and overall tau aggregation in mice (Schaler et al., 2021). GPCRs mediate critical physiological functions and are considered the most successful drug targets (34% of all FDA-approved drugs) prescribed for a broad spectrum of diseases (Hauser et al., 2017). The class of Gs-coupled GPCRs that mediate synapse-to-nucleus signaling by stimulating the AC/cAMP/PKA/CREB pathway has many therapeutic potentials specifically for synaptogenesis and synaptic plasticity (Teich et al., 2015; Fisher et al., 2016).

The GPCRs of the serotonergic innervation are considered the largest group of receptors found in mammals, with wide expression in the CNS and periphery (Smith et al., 2017). Of the 14 serotonin receptor subtypes identified in mammals, 5-HT4R is strongly linked to AD pathology, synaptic plasticity, and neuroprotection (Hannon and Hoyer, 2008). 5-HT4R is mainly an excitatory GPCR localized post-synaptically and is expressed in the brain areas involved in cognition and emotion, such as the hippocampus, prefrontal cortex, amygdala, and basal ganglia (Beliveau et al., 2017). The AD-like effects at the behavioral, cellular, and molecular levels have been reported in a 5-HT4R KO mouse (Karayol et al., 2021). Moreover, the depletion of the serotonergic system and 5-HT4R is evident in post-mortem AD brains (Reynolds et al., 1995).

A large body of animal studies has shown that 5-HT4R agonists appear to be central to enhanced memory function by synaptic plasticity-related enhancement. The activation of 5-HT4R stimulates adenylate cyclase (AC) to produce cAMP, which then the signal is amplified by the activation of PKA, which is known to enhance the cAMP response element-binding protein (CREB)-mediated transcription. Furthermore, activation of 5-HT4R leads to the release of the non-amyloidogenic N-terminal ectodomain of APP, known as sAPPα, which has a potent memory-enhancing effect by displaying neuroprotective and neurotrophic properties (Hashimoto et al., 2012). Importantly, 5-HT4R has been a successful drug target for the intestine to promote intestinal peristalsis (Lanthier et al., 2020). However, early FDA-approved agonists have had adverse cardiovascular events. The latest FDA-approved 5-HT4R agonist, prucalopride, is highly selective, has an excellent safety profile, and has no adverse cardiovascular effects (Yiannakou et al., 2015).

In this study, we tested whether two highly selective 5-HT4R partial agonists, prucalopride and RS-67333, can enhance tau clearance in synapses via proteasome and attenuate tauopathy in PS19 mice. Furthermore, our transcriptomics analyses from AD postmortem brains show that transcripts related to the 5-HT4R signaling cascade were reduced in neurons but not in other cells across Braak stages and that the levels of 5-HT4R inversely correlated with Aβ plaques.

## Materials and methods

### Study design

The goal of our study was to investigate the therapeutic effect of 5-HT4 stimulation on tauopathy mice, the PS19 mouse model. Testing if the stimulation of 5-HT4 by the FDA-approved drug prucalopride and a well-studied small molecule RS-67333 lead to reduced synaptic tau by enhancing proteasome degradation capacity and attenuating tauopathy. To this end, we designed our *in vivo* studies by using PS19 and wild type (WT) mice aged 5–6 months, which were randomly assigned to three experimental groups: vehicle, prucalopride (3 mg/kg; Sigma # 1371) (Ishii et al., 2019), and RS-67333 (2 mg/kg; Sigma # 1882) (Mendez-David et al., 2014); and treated twice daily intraperitoneally for 6 weeks. The drugs were dissolved in the vehicle (0.9% NaCl and 1% DMSO). Drug administration and testing were performed in three sets of mice (two sets of PS19 mice and one set of WT mice). Vehicle (*n* = 21), prucalopride (*n* = 18), and RS-67333 (*n* = 19) in the PS19 mouse group. And vehicle (*n* = 11), prucalopride (*n* = 12), and RS-67333 (*n* = 9) in the wild-type mouse group. The behavioral tests were conducted blinded to treatment group allocation. Confocal microscopy and image analysis were performed blinded to treatment conditions and genotype. The number of biological replicates for each *in vivo* and *in vitro* experiment is specified in the figure legends. D’Agostino and Pearson or Shapiro–Wilk normality tests were used to assess normality. Animal protocols and procedures were approved by the Committee on the Ethics of Animal Experiments of Columbia University Irving Medical Center (CUIMC). They were in full compliance with the U.S. National Institutes of Health Institutional Animal Care.

### Mice

The heterozygous PS19 (P301S) transgenic line and the WT mice with the same background (B6C3) were used with equal male and female mice. The transgene is driven by the murine prion protein (Prnp) promoter and expresses the P301S human 1N4R tau isoform. Mice were housed in pathogen-free conditions under a normal 12-h light/dark cycle.

### Intraperitoneal injections

PS19 and WT mice aged 5–6 months were randomly assigned to three experimental groups: vehicle, prucalopride (3 mg/kg) (Ishii et al., 2019), and RS-67333 (2 mg/kg) (Mendez-David et al., 2014); and treated twice daily intraperitoneally for 6 weeks with the vehicle and 5-HT4R highly selective partial agonists; prucalopride (Sigma, cat# 1371) or RS-67333 (Sigma, cat# 1882). The drugs were dissolved in the vehicle (0.9% NaCl and 1% DMSO). Drug administration and testing were performed in three sets of mice (two sets of PS19 mice and one set of WT mice). Animal experiments were in full compliance with the U.S. National Institutes of Health Institutional Animal Care and Use Committee guidelines and overseen by Columbia University Irving Medical Center.

### Immunofluorescence

Mouse brains were isolated after transcardial perfusion with PBS, and half of the brains were drop-fixed in 4% PFA overnight and then subjected to cryoprotection treatment in 30% sucrose in PBS for 24 h. The other hemisphere was used for homogenization assays. Free-floating brain sections (50 μm) from brains sectioned in the sagittal plane were used. The sections were incubated at 4°C overnight with primary antibody diluted in PBS containing 0.3% Triton X-100 and 5% normal goat serum blocking solution (Vector Laboratories, #S-1000). Anti-mouse monoclonal pS202/pT205 1:1,000 (AT8, 1:500, #MN, 1020 from Thermo Fisher) was used. Following washes, sections were incubated with goat anti-mouse IgG Alexa 594 (Thermo Fisher #A-11005, 1:500). Nine animals (three slices each) per treatment were used. Staining was visualized by confocal microscopy, Zeiss LSM710 confocal microscope at 20× dry objective. Sequential tile scans were performed to capture images of 1,024 × 1,024 resolution. All images from the same experiment were taken at the same laser intensity and detector gain. The immunofluorescence signal for phosphorylated tau was quantified by Fiji-2/ ImageJ software via a macro developed in our lab. Computed values for the total area indicate the intensity of the fluorescence signal. The average of three slices per brain with *n* = 9/condition were used. Tabulated values were normalized before using GraphPad prism to create graphical data representations.

### Kinetic assay for proteasome activity

Cortical brain tissue was harvested and homogenized in a buffer containing 50 mM Tris–HCl (pH 7.4), 5 mM MgCl_2_, 5 mM ATP, 1 mM dithiothreitol, 1 mM EDTA, 10 mM NAF (sodium fluoride), 25 mM β-glycerolphosphate, phosphatase inhibitors, and 10% glycerol, which preserved 26S proteasome assembly and centrifuged at 20,000 × *g* for 25 min at 4°C. The supernatant was normalized for protein concentration determined by Bradford assay. The kinetic assay was used to measure proteasome activity. Samples (lysate, 25 μg per well) and proteasome substrate, 100 μM Suc-LLVY-amc, were mixed. Suc-LLVY-amc is a pentapeptide linked to a fluorogenic signal which, when released by the proteolytic cleavage by the proteasome, emits a fluorescence signal (Ex. 380 nm; Em. 460). For kinetic measurement of the fluorescence signal, we used the Tecan Spark multimode microplate reader (Tecan Inc.), equipped with automated functions to measure the signal over time, for 2 h every 2.5 min. The rate of fluorogenic signal is proportional to the proteasome activity.

### Tissue fractionation and protein extraction

Frozen hemispheres free of cerebellum and brainstem were weighed and homogenized without thawing in RIPA buffer (10× volume/weight) [50 mM Tris–HCl, pH 7.4, 1% NP-40, 0.25% sodium deoxycholate, 150 mM NaCl, 1 mM EDTA, 1 mM phenylmethylsulfonyl (PMSF), 1 mM sodium orthovanadate, 1 mM sodium fluoride (NaF), 1 μl/ml protease inhibitor mix]. Homogenates were centrifuged for 10 min at 3,000 × *g* at 4°C. Protein assay was performed on the clear supernatants representing the total extract used to analyze the total protein levels. Sample volumes were adjusted with RIPA buffer containing 100 mM DTT and NuPAGE LDS Sample Buffer 4× buffer (Life Technologies) and boiled for 5 min. The sarkosyl-insoluble extracts, which are highly enriched in aggregated tau species, were generated when 250 μg aliquots from the total protein extracts were normalized into 200 μl final volume containing 1% sarkosyl, followed by ultra-centrifugation at 100,000 × *g* for 1 h at 4°C. Without disturbing the pellet, the supernatant was transferred to new tubes. The pellet was resuspended in 100 μl RIPA buffer containing DTT and NuPAGE LDS Sample Buffer 4× buffer, followed by vortexing for 1 min and 5 min heating at 95°C.

### Immunoblot analysis

Samples (2.5–10 μg protein) were typically run on precast 4%–12% Bis-Tris gels (Life Technologies; WG1403BOX10) using MOPS buffer (NP0001). Proteins were analyzed after electrophoresis on SDS-PAGE and transferred onto 0.2-μm nitrocellulose membranes. Samples were run independently for each protein. I.e., blots were not stripped and re-probed with different antibodies as most proteins had similar molecular weights. Blots were blocked and incubated with primary and secondary antibodies at the below concentrations. Membranes were developed with enhanced chemiluminescent reagent [Immobilon Western HRP substrate and Luminol reagent (WBKLS0500, Millipore)] using a Fujifilm LAS3000 imaging system. ImageJ^1^ was used to quantify the signal. Relative intensity (fold change or fold increase, no units) is the ratio of the value for each protein to the value of the respective loading control.

### Antibodies

Monoclonal human tau (CP27; 1:5,000) and pS396/pS404 (PHF1; 1:2,500) were generous gifts from the late Dr. Peter Davies. Mouse monoclonal pS202/pT205 Tau (AT8, 1:2,500, #MN, 1020) was from Thermo Fisher. Monoclonal anti-rabbit K-48 linked ubiquitin (1:1,000, D905) from Cell Signaling. Mouse monoclonal anti-PSD-95 (1:5,000, 6G6-1C9, ab2723); rabbit monoclonal synaptophysin (YE269, ab32127, 1:5,000) were from Abcam. Loading control anti-actin (1:5,000 AC-74, #A2228) was from Sigma. Secondary antibodies were from Jackson Immunoresearch, anti-mouse (115-035-003), and anti-rabbit (111-035-003).

### Synaptic fractions

#### Fractionation assay

Four hemi cortices/experiment from mice (2 hemi cortices/gender) were gently homogenized in ice-cold homogenization buffer (320 mM sucrose made in hypotonic buffer, 25 mM HEPES-KOH pH 7.5, 1 mM EDTA, 5 mM MES pH 7.5, 5 mM MgCl_2_, 5 mM ATP pH 7.5, 1 mM DTT, 10 mM NAF, 25 mM βglycerol phosphate, phosphatase, and protease inhibitors). The homogenates were centrifuged at 500 × *g* for 5 min at 4°C. The supernatant (total extract) was further centrifuged at 19,000 × *g* for 20 min at 4°C. The resulting supernatant is a cytosolic fraction, and the pellet is a crude synaptosome resuspended in homogenizing buffer.

#### Discontinuous sucrose density gradient

Crude synaptosome extracts were layered on top of a nonlinear sucrose gradient (1.2 M, 0.8 M, and 0.32 M sucrose from bottom to top) and centrifuged at 300,000 × *g* for 4 h at 4°C. After which, synaptosomes sediment at the interface between 1.2 M and 0.8 M sucrose layer. To separate synaptosomes into pre and post-synaptic fractions, collected synaptosomes (∼3–4 ml/sample) were diluted in 0.01 mM CaCl_2_, 20 mM Tris–HCl pH 6, 1% Triton X-100, with protease and phosphatase inhibitors and mixed by inversion for 20 min at 4°C. After incubation, samples were centrifuged at 40,000 × *g* for 20 min. The resulting pellet was collected as a post-synaptic fraction, and the supernatant was collected as a pre-synaptic fraction. After separating pre and post-synaptic compartments, fractions were precipitated in ice-cold acetone and concentrated by Amicon-15 10 kDa cut-off tubes. Both fractions were resuspended in PBS with 0.05% Triton X-100 with protease/phosphates inhibitors and sonicated. The fractionation assay was repeated at least three times. Protein concentration was determined by Bradford assay. Samples were diluted to 0.5 μg/μl and 2.5 μg/well loaded for Western blotting. Immunoblots for each protein were run independently at an equal amount of proteins. For quantification, the relative values of three independent fractionation experiments from the vehicle groups were normalized to 100% and were compared to the treatment groups. The values were then plugged into GraphPad Prism 9 software to generate bar graphs with SEM.

### Nest building test

The nest-building behavior has been used to assess mice cognition. PS19 or WT mice were singly housed and were given intact compressed cotton nestlets in the center of the cage. After 24 h, images were taken for each nest from corresponding mice. Nest scoring used a 1–5 scale, as described previously (Neely et al., 2019). Scores: 1 – untouched nestlet; 2 – partially torn nestlet, 3 – partially complete nest, 4 – nest was almost formed, 5 – nest complete. All mice were tested after 6 weeks of treatments.

### Open field test

The Open Field is the most commonly used test for spontaneous exploratory activity in a novel environment, incorporating measurements of locomotion and anxiety-like behaviors. The Open Field test was performed following previously described protocols (Cho et al., 2021). Exploration was monitored during a 60 min session with Activity Monitor Version 7 tracking software (Med Associates, Inc.). Briefly, each mouse was gently placed in the center of a clear Plexiglas arena (27.31 × 27.31 × 20.32 cm, Med Associates ENV-510) lit with dim light (∼5 lux), and is allowed to ambulate freely. Infrared (IR) beams embedded along the *X*, *Y*, and *Z* axes of the arena automatically track distance moved, horizontal movement, vertical movement, stereotypies, and time spent in center zone. Data are analyzed in six, 10-min time bins. Arenas are cleaned with 70% ethanol and thoroughly dried between trials.

### Human transcriptomic analyses of HTR4/AC/PKA/CREB1 pathway-related genes

All the human datasets for bulk RNA transcriptomic data on AD were accessed from the AD Knowledge Portal.^2^ We examined the three cohorts, Mount Sinai Brain Bank (MSBB), Religious Orders Study/Memory and Aging Project (ROSMAP), and Mayo study. MSBB includes 299 individuals with multiple brain region tissue samples (214 parahippocampal gyrus samples, 261 frontal pole samples, 221 inferior frontal gyrus samples, and 240 superior temporal gyrus samples), and ROSMAP includes 633 individual dorsolateral prefrontal cortex samples. Mayo study comprises 355 individuals with 319 temporal cortex (TCX) and 278 cerebellum samples. Because the cerebellum is less affected in AD, especially in the early stage, the 278 cerebellum samples from Mayo were removed from the examination.

Raw transcriptomic count data were normalized with variance stabilizing transformation with DESeq2 (Love et al., 2014). The effects of HTR4/AC/PKA/CREB1 pathway-related gene expression levels on the degree of tau aggregation, which was categorized by Braak scores, were examined with ordinal regression after adjusting for the confounding effects. For MSBB, the confounding effects from age, gender, ethnicity, post-mortem interval (PMI), RNA integrity number (RIN) and library size were adjusted. For ROSMAP, the confounding effects from age, gender, ethnicity, education, PMI, RIN, and library size were adjusted. For Mayo TCX samples, because all the samples were from the white population, the confounding effects from age, gender, RIN, and library size were adjusted. The false discovery rate (FDR) approach was applied to multiple testing corrections. Then meta-analyses were performed to integrate the regression results across the three cohorts on individual genes with R package meta (Balduzzi et al., 2019).

### Human single nuclei RNA-seq analyses of HTR4R/AC/PKA-CREB1 pathway-related genes

Single nucleus RNA-seq (snRNA-seq) data from Mathys et al. (2019), which consists of 80,660 single-nucleus transcriptomes from the prefrontal cortex (PFC) of 48 individuals from ROSMAP, was accessed from the AD Knowledge Portal snRNA-seq data from Seattle Alzheimer’s Disease Brain Cell Atlas (SEA-AD), which is comprised of 1,240,908 single-nucleus transcriptomes from the middle temporal gyrus (MTG) of 84 individuals, was accessed from Chan Zuckerberg Initiative Science.^3^

Raw snRNA-seq count data were normalized with log2 (TP10K + 1), where TP10K, or transcripts per 10,000, is the RNA-seq quantification measure that accounts for differences in sequencing depth across cells. A mixed-effect regression model with individual samples as a fixed effect is not applicable to examine the effect of the expression of genes on the degree of tau aggregation due to the dropouts in snRNA-seq (Qiu, 2020). To determine whether the expression of HTR4/AC/PKA/CREB1 pathway-related genes differs among different degrees of tau aggregation for each cell type, a non-parametric one-way analysis of variance (ANOVA) and Kruskal–Wallis test was applied followed by *post-hoc* pairwise comparisons with Wilcoxon rank sum test. The FDR approach was applied to multiple testing corrections.

### Statistical and bioinformatics analyses

**FIGURE 1:**
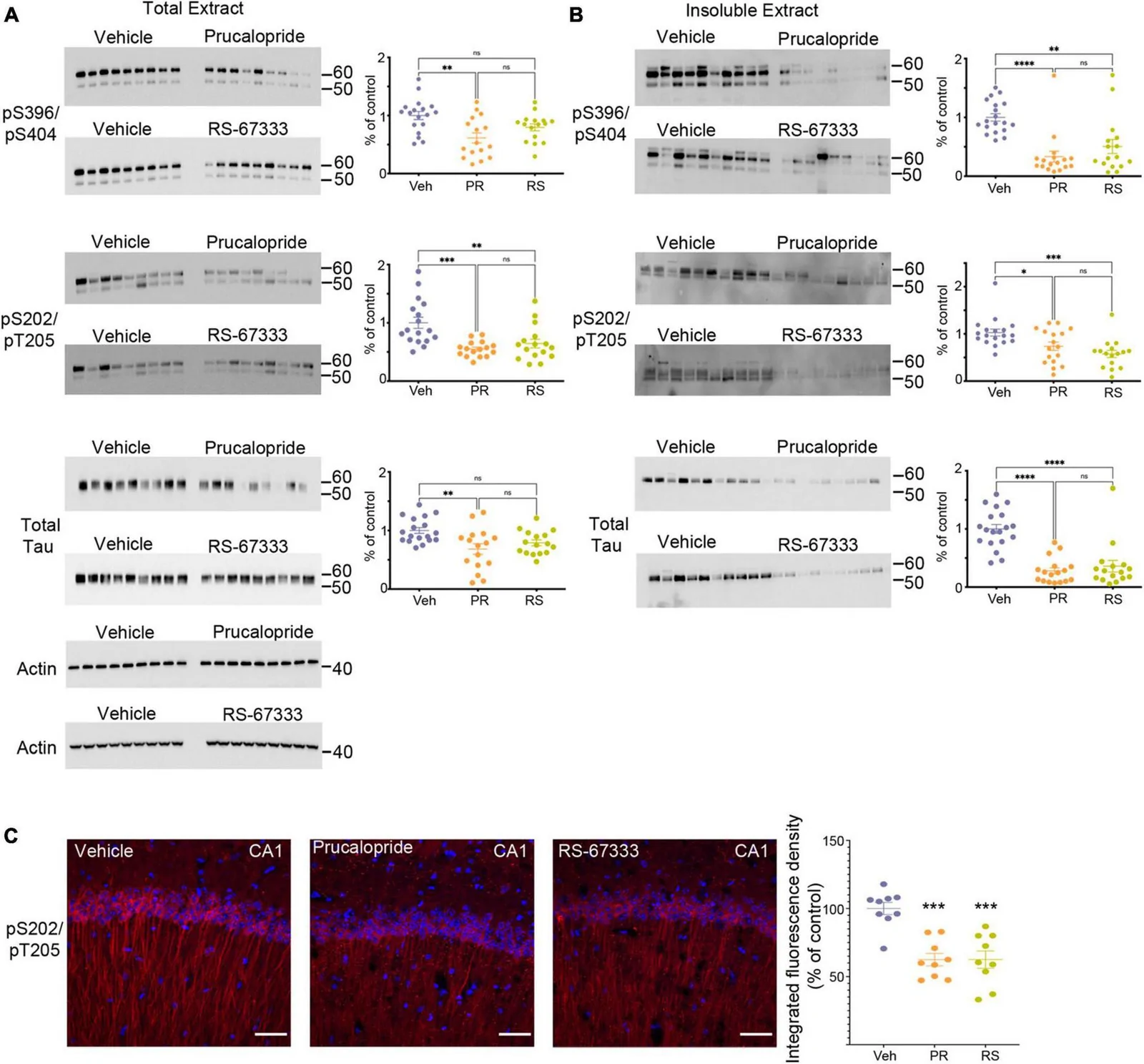
Stimulation of 5-HT4R by prucalopride or RS-67333 attenuates tau pathology in PS19 mice. Representative immunoblots and corresponding densitometric quantification for pS396/pS404, pS202/pT205 tau epitopes and total tau from **(A)** total extracts and **(B)** insoluble extracts from brains of PS19 mice treated with vehicle (Veh), prucalopride (PR) or RS-67333 (RS). Quantified densitometry for immunoblots expressed as percent relative to vehicle-treated mice. Vehicle treated animals were compared with prucalopride or RS-67333. **(C)** Immunofluorescence labeling of pS202/pT205 tau epitope (red) and DAPI (blue), and quantification of integrated fluorescence density for pS202/pT205 tau epitope in the CA1 region of the hippocampus of PS19 mice treated with vehicle, prucalopride or RS-67333. Scale bar, 100 μm. Scatter plots represent the quantification of immunoreactivity normalized to actin. Statistical analyses of vehicle (*n* = 19), prucalopride (*n* = 17), and RS-67333 (*n* = 16) mice were performed in two sets (this figure and Supplementary Figures 1, 2). Quantification of immunofluorescence intensity used three brain slices from nine animals/groups. For statistical analyses, we used one-way ANOVA followed by Bonferroni multiple comparison *post-hoc* tests. Error bars mean ± SEM. ^ns^Not significant, *P < 0.05, **P < 0.01, ***P < 0.001, and ****P < 0.0001.

**FIGURE 2:**
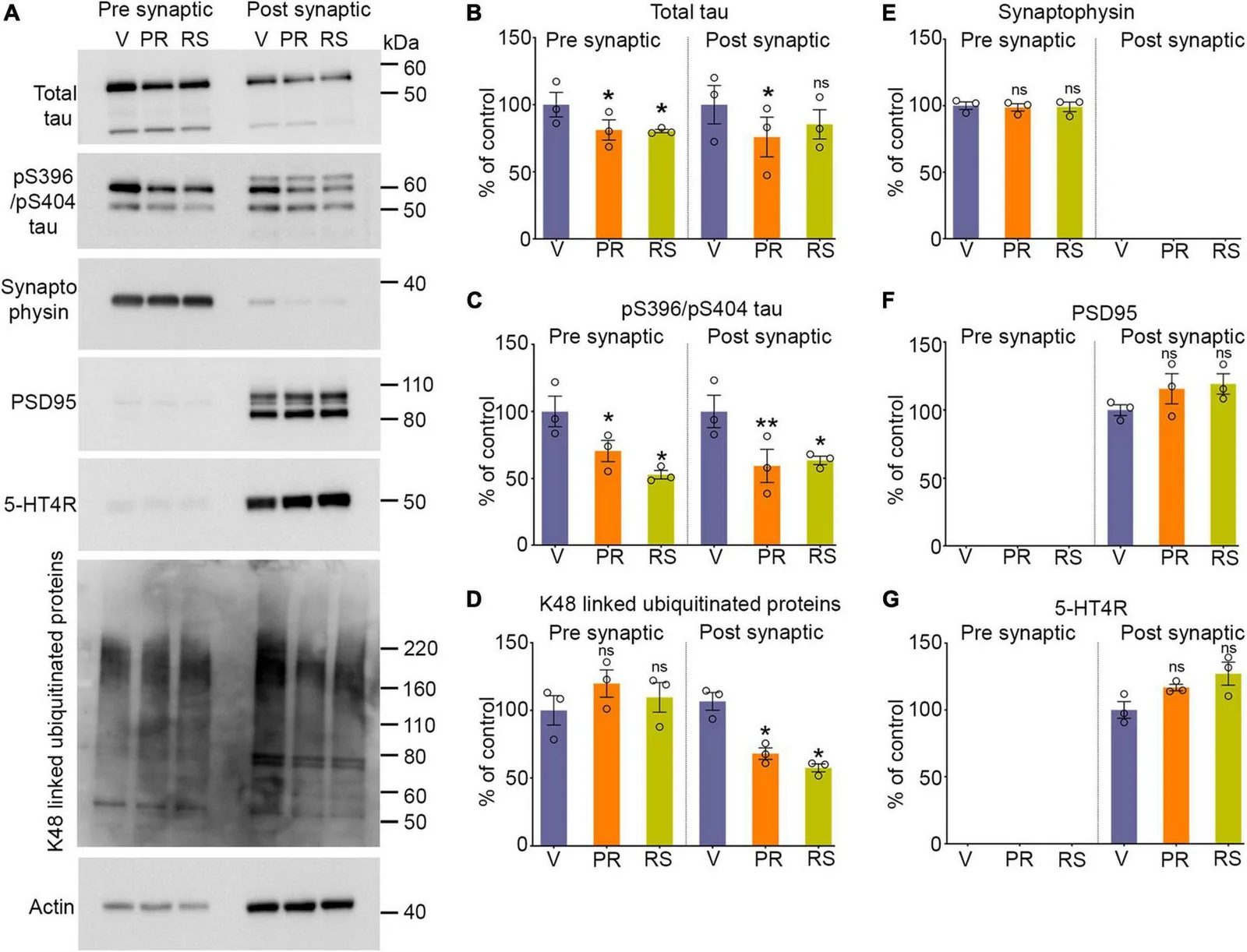
Stimulation of 5-HT4R by prucalopride or RS-67333 reduces synaptic tau and ubiquitinated proteins in PS19 mice. **(A)** Representative immunoblots of pre-synaptic and post-synaptic fractions from the brains of PS19 mice treated with vehicle (V), prucalopride (PR), or RS-67333 (RS). Blots were probed for total tau, pS396/pS404, and pS202/pT205 tau epitopes, K48-linked ubiquitinated proteins, for pre-synaptic (synaptophysin) and post-synaptic (PSD95) markers, and 5-HT4R. Actin was a loading control and was used for normalization. **(B–G)** Quantified densitometry for immunoblots, expressed as percent relative to vehicle-treated mice. Three fractionation experiments were performed, and for each fractionation experiment, four mouse hemi-cortices were pooled together (overall, *n* = 12 hemicortices per treatment). One-way ANOVA followed by Bonferroni, multiple comparisons post hoc tests, was conducted. Quantified results from prucalopride and RS 67333 and were compared with vehicle treated littermates. Data are presented as means ± SEM; ns, not significant, *P < 0.05, **P < 0.01.

**FIGURE 3:**
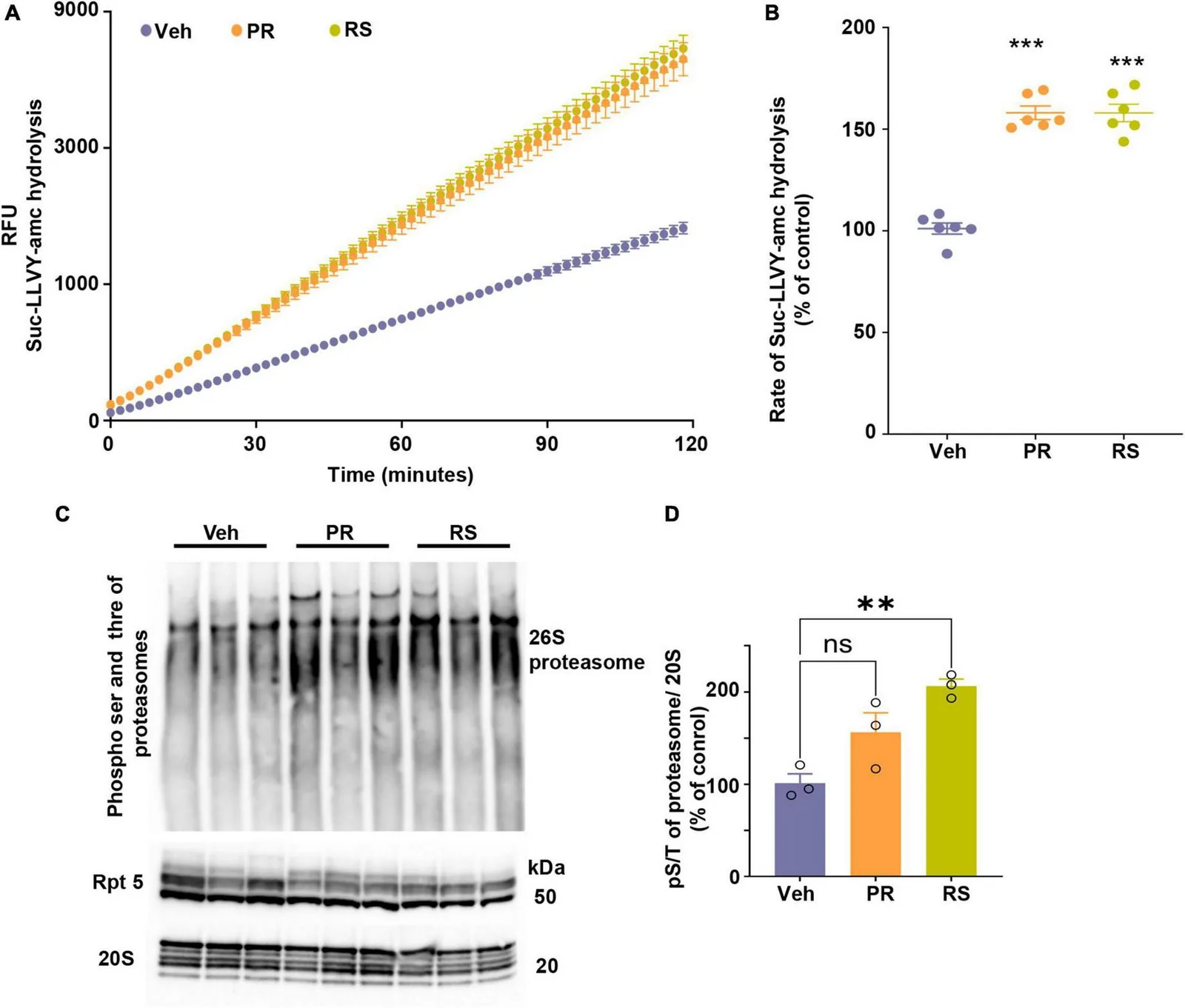
Stimulation of 5-HT4R by prucalopride or RS-67333 enhances proteasome activity in the brain of PS19 mice. **(A)** The peptidase activity of proteasomes from the total brain extracts of PS19 mice treated with vehicle (V), prucalopride (PR), or RS-67333 (RS) was assessed for 120 min using the fluorogenic substrate Suc-LLVY-amc (40 μM). *Y*-axis represents relative fluorescence units (RFU). **(B)** The graph represents the rate of substrate hydrolysis by proteasomes from panel **(A)** expressed as a percentage of vehicle control. Total proteasome lysate from *n* = 6 animals/treatment was used in the assays, and three independent proteasome kinetic assays were performed. **(C)** Native gel was immunoprobed for PKA-specific phospho S/T and samples were run also for Rpt5 and 20S proteasome subunits. **(D)** Quantification of hyper phosphorylation of 26S proteasome normalized to 20S α1-7 subunits of the proteasomes. One-way ANOVA, followed by Bonferroni multiple comparison post hoc tests, was used for panels **(B,D)**. Error bars mean ± SEM. ^ns^Not significant (*p* = 0.059), **P < 0.01, ***P < 0.001.

**FIGURE 4:**
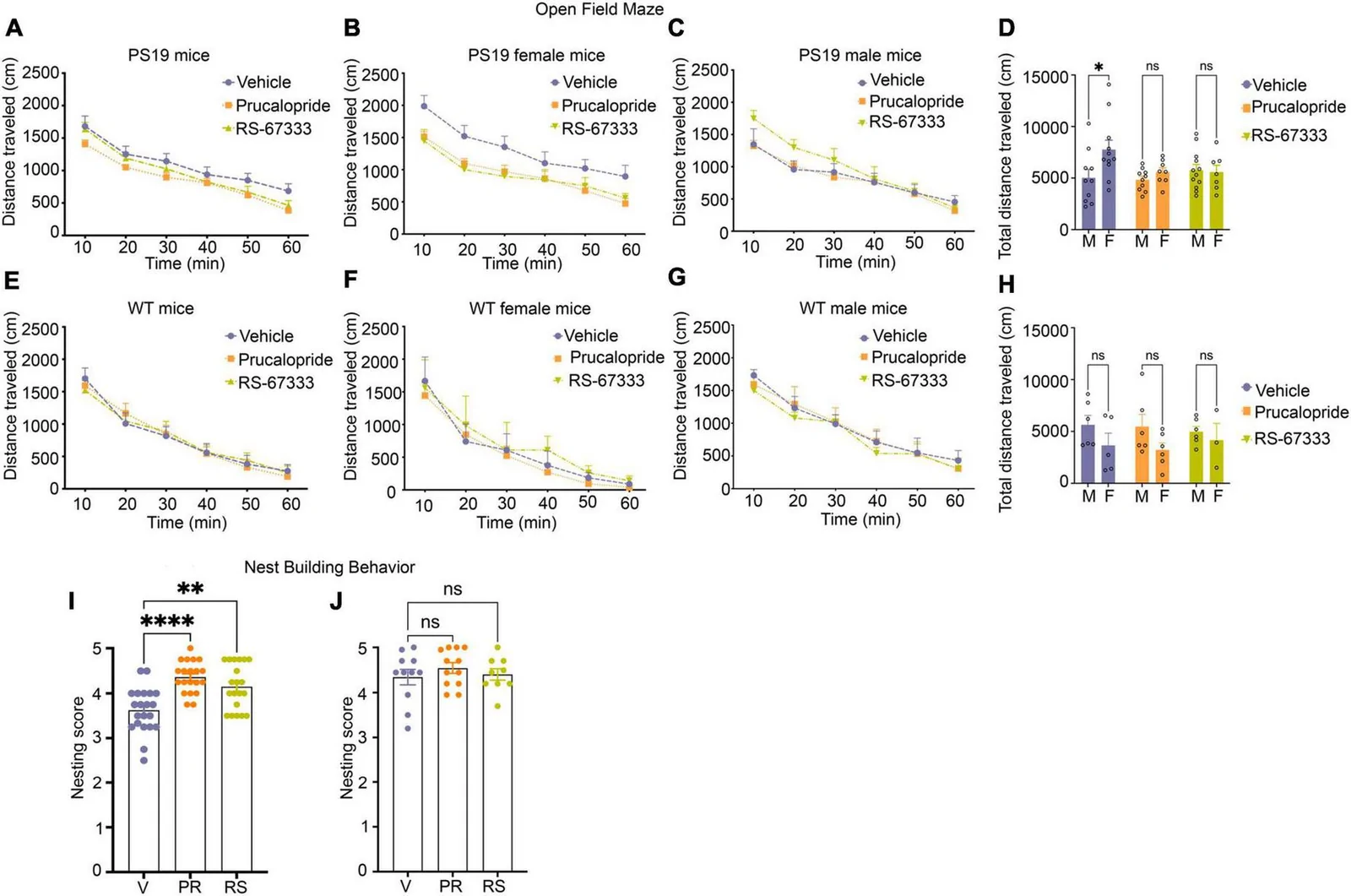
Stimulation of 5-HT4R by prucalopride or RS-67333 reduces anxiety-related behavior and improves cognitive performance in early-stage tauopathy in PS19 mice. **(A–H)** The open field test was used to assess locomotor activities and anxiety-related behaviors by measuring the ambulatory distance over 60 min. PS19 and wild-type mice were treated with vehicle, prucalopride, or RS-67333. Vehicle (*n* = 21), prucalopride (*n* = 18), and RS-67333 (*n* = 19) in the PS19 mouse group, and vehicle (*n* = 11), prucalopride (*n* = 12), and RS-67333-(*n* = 9) in the wild-type mouse group. **(A)** The traveled distance of PS19 mice showed no significant changes due to the treatment effect. **(B,C)** Grouped by sex and treatment. **(B)** Vehicle-treated females showed significantly longer total ambulatory distance compared to female littermates treated with prucalopride or RS-67333. When compared across 10 min bins; the significance was reached in the first 10 and 20 min bins between vehicle and RS-67333 treated female PS19 littermates. **(C)** Male littermates showed no changes in ambulatory distance due to treatment. **(D)** Total ambulatory distance in a vehicle, prucalopride, or RS-67,333 treated groups separated by sex, shows that vehicle-treated female PS19 mice exhibit significantly higher ambulatory distance compared to their vehicle-treated male littermates. Whereas prucalopride or RS-67333 treatment normalized the total distance of the female group to the distance traveled by male littermates. **(E–H)** Wild-type (WT) mice showed no difference in ambulatory distance in sex and treatment effect across the three groups. The open-field test data were analyzed using a two-way repeated-measures ANOVA with *post hoc* Bonferroni correction. **(I,J)** Nest building test for PS19 and WT mice male and female together. **(I)** Nesting scores from vehicle (*n* = 21), prucalopride (*n* = 18), and RS-67333 (*n* = 19) in PS19 mice male and female. **(J)** Nesting scores from vehicle (*n* = 11), prucalopride (*n* = 12), and RS-67333 (*n* = 9) in WT mice male and female. Nest building test scores were assigned 24 h after one compressed cotton nestlet was placed in each cage. Nesting data were analyzed using the non-parametric Kruskal–Wallis test. Data are reported as means SEM. ns, not significant, *P < 0.05, **P < 0.01, ****P < 0.0001.

For mouse data, statistical analyses for Figures 1–4 were performed with Prism 9 (GraphPad Software, San Diego, CA, USA). *P* < 0.05 was considered significant. Data were assessed for normality using the Shapiro–Wilk test used to determine the homogeneity of variance. Data for Figures 1, 3 were analyzed using one-way ANOVA with Bonferroni *post hoc* correction. Data for Figure 2 were analyzed with one-way and two-way ANOVA with *post hoc* Bonferroni correction. Data for Figure 4 (OF test) were analyzed with two-way repeated measure ANOVA with *post hoc* Bonferroni correction. Data for nest building results (Figure 4) were analyzed with the Kruskal–Wallis test with multiple comparisons of Dunn’s correction.

**FIGURE 5:**
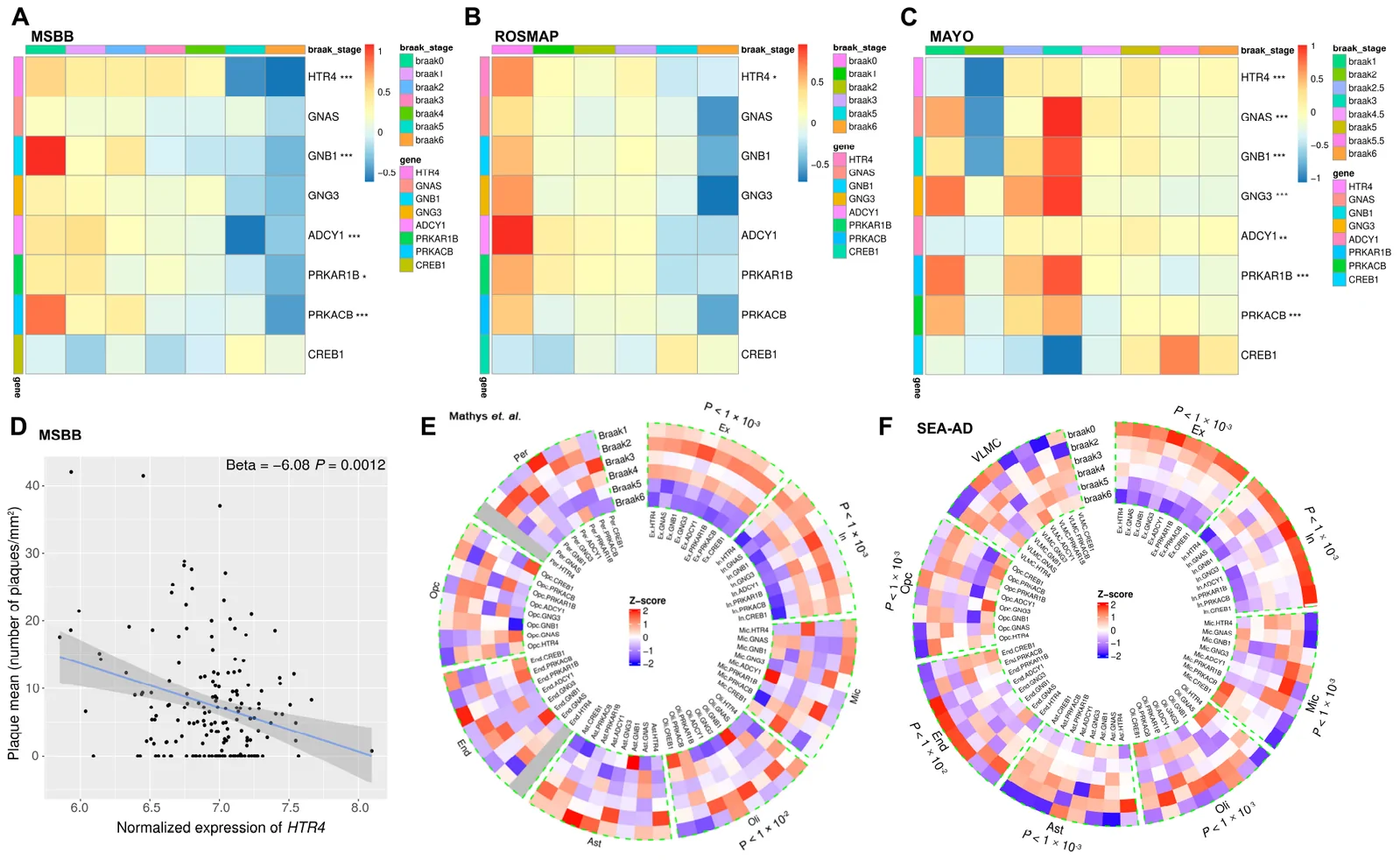
The RNA-seq analyses show decreased expression of 5-HT4R/AC/PKA/CREB1 pathway-related genes with exacerbated tau aggregation. **(A–C)** Bulk RNA-seq data analyses presented as heatmaps showing HTR4/AC/PKA/CREB1 pathway-related genes (except CREB1) were decreased in advanced Braak stages in the AD post-mortem brains. Brain areas **(A)** parahippocampal gyrus (PHG) from MSBB study. **(B)** Dorsolateral prefrontal cortex from the ROSMAP study. **(C)** Temporal cortex from the Mayo study. Gene expressions were scaled with *Z*-score transformation. Ordinal regression was used to assess the correlation between Braak stages and gene expression after adjusting for the effects of confounding factors such as age, gender, ethnicity, PMI, RIN, and library size. *P < 0.05, **P < 0.01, and ***P < 0.001 after FDR correction. **(D)** Scatter plot showing the HTR4 expression was negatively correlated with Aβ plaque measure in PHG from MSBB study with linear regression after adjusting for confounding effects. **(E,F)** Single nucleus RNA-seq analyses presented in circular heatmaps show the expression percent of HTR4/AC/PKA/CREB1 pathway-related genes were decreased with advancing Braak stages in excitatory and inhibitory neurons. **(E)** Prefrontal cortex from Mathys et al. (2019) dataset study. **(F)** The middle temporal gyrus from SEA-AD studies and Gene expression percentages were scaled with *Z*-score transformation. SnRNA-seq data were analyzed using the Kruskal–Wallis test; *P < 0.05, **P < 0.01, and ***P < 0.001 after FDR correction. Ex, excitatory neurons; In, inhibitory neurons; Mic, microglia; Oli, oligodendrocytes; Ast, astrocytes; End, endothelial cells; Opc, oligodendrocyte progenitor cells; Per, pericytes; VLMC, vascular leptomeningeal cells.

Statistical and bioinformatics analyses for human data for Figure 5 and Supplementary Figure 3 were performed with R (version 4.2.2) and Python (version 3.9.13). For Figures 5A–C, the effects of HTR4/AC/PKA/CREB1 pathway-related gene expression levels on the degree of tau aggregation were examined with ordinal regression after adjusting for the confounding effects, such as age, gender, ethnicity, PMI, RIN, and library size, with *post hoc* FDR correction. For Figure 5D, the effect of HTR4 gene expression on Aβ plaque measure in parahippocampal gyrus (PHG) from the MSBB study was examined with linear regression after adjusting for the confounding effects from age, gender, ethnicity, PMI, RIN, and library size. For Figures 5E, F, due to the zero inflation introduced by undetectable dropouts in snRNA-seq, mixed-effect regression model was not applicable. Instead, we examined the differences of HTR4/AC/PKA/CREB1 pathway-related gene expression among different degrees of tau aggregation for each cell type with Kruskal–Wallis test followed by *post-hoc* pairwise comparisons with Wilcoxon rank sum test with FDR correction. We used a relatively more liberal multiple test correction than the Bonferroni used in mouse data because of large sample sizes in bulk-seq data and large cell counts in snRNA-seq data. The heatmaps for Figures 5A–C were generated with pheatmap (version 1.0.12). The scatter plot for Figure 5D was generated with ggplot2 (version 3.4.0). The circular heatmaps for Figures 5E, F were generated with circlize (version 0.4.15). The meta-analysis forest plots for Supplementary Figure 4 were generated with meta (version 6.0.0).

## Results

### Chronic 5-HT4R stimulation reduced tau pathology

To investigate the effect of 5-HT4R stimulation on tauopathy, we used two selective, high-affinity 5-HT4R partial agonists with good brain penetration (Johnson et al., 2012), prucalopride (3 mg/kg) and RS-67333 (2 mg/kg). Prucalopride is an FDA-approved medication for treating constipation with an excellent safety profile and has recently been shown to exert a pro-cognitive effect and increase hippocampal function in a small clinical trial in healthy subjects (de Cates et al., 2021). In animal models, RS-67333 was extensively shown to have pro-cognitive (Quiedeville et al., 2015) and anxiolytic/anti-depressant-like activity (Mendez-David et al., 2014).

Adult 5–6 months old PS19 or WT mice were treated intraperitoneally twice daily for 6 weeks. At this age, PS19 mice develop early-stage tauopathy. After behavioral studies, cortical and hippocampal tissue was collected to generate the total and insoluble extracts to assess the hyperphosphorylated and aggregated tau species and to analyze the results by quantitative immunoblotting. Our data show that prucalopride had a significant effect in reducing phosphorylated and total tau forms in the total (Figure 1A) and the insoluble brain extracts (Figure 1B), likewise RS-67333 showed a similar effect (Figures 1A, B), albeit a non-significant trend was detected in the total extract for pS396/pS404, and total tau forms (Figure 1A). Complete Western blotting are shown in Supplementary Figures 1, 2. Interestingly, stimulation of 5-HT4R in WT littermates lead to market reduced of murine tau in prucalopride and RS-67333 (Supplementary Figure 3). Showing that even in healthy conditions 5-HT4 stimulation can lead to reduced normal tau. Immunohistochemical analyses of the hippocampus, shown here in the CA1 region, also showed that 5-HT4R agonists markedly reduced pS202/pT205 tau epitope (Figure 1C).

### 5-HT4R stimulation reduced synaptic tau

Synaptic tau accumulation and trans-synaptic propagation of pathological tau contribute to synaptic degeneration and cognitive deficits in AD and other tauopathies. We have shown previously that post-synaptic accumulation of tau in the early stages of the disease is toxic and seed competent, able to template and cause aggregation of naïve endogenous tau before the NFT appear in the brain (Schaler et al., 2021). Moreover, combating the accumulation of tau in the post-synaptic compartment by enhancing the turnover of tau restricted to synapses by the proteasome leads to reduced tauopathy (Schaler et al., 2021). In this study, we tested whether stimulation of 5-HT4R found predominantly on the surface of post-synaptic compartments can enhance proteasome activity to clear synaptic tau. We isolated gradient-purified synapses and subsequently separated isolated synapses into pre and post-synaptic fractions from the vehicle, prucalopride, and RS-6733 treated PS19 mice to detect tau changes within synapses by quantitative immunoblotting. Prucalopride treatment significantly decreased the total and pS396/pS404 tau in the pre and post synaptic compartments compared to the vehicle-treated group (Figures 2A–C). Similarly, RS-67333 showed a significant decrease in the pS396/pS404 tau in both synaptic compartments compared to vehicle treated group. Total tau was significantly decreased in the pre-synaptic fraction and showed a non-significant trend decrease in the post-synaptic compartment compared to vehicle treated group (Figures 2A–C). While tau species were reduced, we did not detect any changes in the markers of the pre and post-synaptic compartments, synaptophysin (Figures 2A, E) and PSD95 (Figures 2A, F), respectively. In fact, PSD95 levels showed a moderate increase in the treatment group, with prucalopride or RS-67333, compared to vehicle control. We confirmed the presence of 5-HT4R in the post-synaptic compartment as it was reported to be present post-synaptically, and its levels were slightly increased in the agonist treatment, but the increase was non-significant (Figures 2A, G).

Next, we tested the status of K-48-specific ubiquitinated proteins exclusively turned over by the 26S proteasome. Our data show that the K-48 specific ubiquitinated proteins were significantly reduced in the post-synaptic fractions in prucalopride and RS-67333 treatment compared to the vehicle treatment group (Figures 2A, D). The pre-synaptic fractions showed no changes in the ubiquitinated protein amount (Figures 2A, D).

### 5-HT4R agonism enhanced proteasome activity

5-HT4R activation stimulates AC as a primary mode of signal transduction and increases cAMP concentration. The signaling cascade is amplified when cAMP interacts and activates PKA, which is known to enhance gene expression related to synaptic plasticity and neurotrophic factors by phosphorylating transcription factors, such as CREB. Moreover, a new function of PKA-mediated phosphorylation leads to hyperphosphorylation of the 26S proteasomes, conferring enhanced degradation capacity to proteasome complexes to degrade proteins in the cells, especially aggregating-prone proteins such as tau (Myeku et al., 2016), poly-GA (Khosravi et al., 2020), huntingtin (VerPlank et al., 2020), and TDP43 (Lokireddy et al., 2015). Altogether this raises the interest in targeting 5-HT4R, not only for its neurogenic and plasticity-related memory improvement but concomitantly as a mode of enhancing the turnover of misfolded toxic proteins that can initially accumulate within synapses. To this end, we assessed proteasome capacity to degrade fluorogenic substrate kinetically over 120 min (Figure 3A). The slope of the reaction was calculated and showed that 5-HT4R agonists significantly increased the proteasome’s proteolytic capacity over time (Figure 3B). PKA-mediated phospho serine threonine of the 26S proteasome was assessed in a native gel (Figure 3C) showing that hyperphosphorylation of 26S proteasome was evident prucalopride and RS-67333 treated animal groups. However, the significance was reached in the RS-57333 treated group. Levels of α subunits of 20S proteasome were used for normalization and quantification of the signal (Figure 3D).

### 5-HT4R agonism reduced anxiety-related behavior and improved cognitive performance in early-stage tauopathy

PS19 mice exhibit mild behavioral phenotypes. Therefore, many standard behavioral tests do not show any changes in cognitive performance in these mice. This is true for other tauopathy models with mild pathology. Recently a detailed characterization of PS19 mice across ages has shown that hippocampal-dependent impairment in spatial reference memory was only apparent in female mice at age 12 months (Sun et al., 2020). However, at this age, these mice develop hindlimb paralysis due to transgene expression in the spinal cord and die between 10 and 12 months of age (Jankowsky and Zheng, 2017). For our studies, we performed the open field test, which evaluates spontaneous locomotor activities as well as anxiety-like behaviors (Kraeuter et al., 2019), and a non-maternal nest-building test, sensitive to hippocampal damage to evaluate cognition (Sun et al., 2020). PS19 mice from three treatment groups (vehicle, prucalopride, and RS-67333, 6–7 months old) showed a similar decrease in the ambulatory distance over 60 min (Figure 4A). However, when we separated the treatment groups by sex, vehicle-treated females showed significantly increased total ambulatory distance (Figures 4B, D), indicating enhanced hyperactivity, compared to male vehicle-treated PS19 littermates. Treatment with agonists blunted the longer traveled distance in the female group (Figures 4B, D), normalizing the results to the male PS19 littermates (Figures 4C, D), which was similar in all treatment groups. In WT mice (6–7 months old), there was no difference in sex and treatment effect across the three groups (Figures 4E–H).

Furthermore, the non-maternal nest-building performance showed that vehicle-treated PS19 mice exhibited significantly impaired nest-building performance compared to prucalopride and RS-67333 treated groups (Figure 4I). In contrast, the WT mice showed no treatment effect in nest-building performance across the groups (Figure 4J). Although vehicle-treated female PS19 mice exhibited hyperactive locomotor activities compared to male vehicle-treated littermates, they did not perform worse in the nest-building test (data not shown).

### 5-HT4R/AC/PKA/CREB genes are decreased with exacerbated tau aggregation in human post-mortem brains

5-HT4R plays an essential role in processes related to learning and memory mediated by the dominant synapse to nucleus signaling cascade through AC/cAMP/PKA pathway, modulating the expression of plasticity/learning-related proteins such as BDNF, AKT, and CREB (Roux et al., 2021). To assess the mRNA expression levels of the canonical proteins in this cascade (5-HT4, G_s_α, Gβ, Gγ, AC, PKA R, and C subunits and CREB), we performed bioinformatics analyses from bulk and snRNA-seq from available datasets. Bulk tissue sequencings were available from the MSBB study with 299 samples, the ROSMAP study with 633 samples, and the Mayo study with 319 samples. SnRNA-seq were available from Mathys et al. (2019), which comprised 80,660 single-nucleus transcriptomes, and SEA-AD, which comprised 1,240,908 single-nucleus transcriptomes.

Before performing the analyses, we assessed the tissue expression specificities of these genes and chose the gene subtypes with high specificity in the brain (Fagerberg et al., 2014).

RNA-seq of bulk tissues presented by heat map shows that except *CREB1*, all genes in the pathway were decreased predominately in advanced Braak stages consistently in all the three bulk tissue cohorts (MSBB, ROSMAP, and Mayo) (Figures 5A–C). Because multiple brain regions were sequenced for the same individuals in the MSBB cohorts, the PHG, which has an early onset of AD pathology, was chosen to be presented (Figure 5A). Similarly, the other brain regions, such as a frontal pole, inferior frontal gyrus, and superior temporal gyrus, showed the same expression patterns of these genes as in PHG (data not shown). Meta-analysis results by combining the three cohorts are shown in Supplementary Figure 4. Except for *CREB1*, the other genes in the pathway show decreased expression in advanced Braak stages. Furthermore, we examined the relationship between the *HTR4* gene and Aβ plaque load from the MSBB cohort where Aβ plaque load information is available and found a statistically significant negative correlation between the HTR4 expression and the Aβ plaque load (Figure 5D). While consistent, bulk RNA-seq results could reflect tissue degeneration in late stages of the disease. Thus, for our next step analyses, we used snRNA-seq datasets from Mathys et al. (2019) and from the SEA-AD with the aforementioned single nucleus counts, respectively. Due to the undetectable low expression levels of some genes in snRNA-seq, which are known as dropouts, and the relatively low expression of the genes of interest, the gene expression in the single cell heatmaps was represented and visualized as expression percent instead of the mean value (Qiu, 2020; Muus et al., 2021). Our analyses show that the expression percent of all the HTR4 pathway-related genes, including *CREB1*, were significantly reduced with advanced Braak stages in excitatory and inhibitory neurons (Figures 5E, F).

For the non-neuronal cell types, in Mathys et al. (2019) study, the genes showed no specific expression patterns with developing tau pathology (Figure 5E), which may be attributable to low cell counts in the dataset. However, in SEA-AD, which sequenced over one million cells, the selected microglia and astrocyte genes significantly increased with advanced Braak stages (Figure 5F).

By comparing the expression patterns of *CREB1* in bulk RNA-seq and snRNA-seq, it was clear that the increased *CREB1* expression in bulk RNA-seq (Figures 5A–C) in advanced Braak stages were due to the increased expression in microglia, oligodendrocytes, endothelial cells, and oligodendrocyte progenitor which counteracted the decreased expression in neurons (Figures 5E, F).

## Discussion

Synaptic dysfunction and loss due to the accumulation of tau in synapses and subsequent trans-synaptic propagation of tau pathology underlines the importance of designing synaptic therapy approaches to preserve synapses and halt the spread of tauopathy and memory decline in AD. The synaptic abnormalities and dysfunction occur in the early stage before the symptomatic stage of the disease. Therefore, it is crucial to understand the mechanisms underlying synaptic damage to help find effective therapies to improve the memory of AD patients.

To develop an effective therapeutic approach to target synaptic tau in the early stages of the disease, we used a receptor-mediated enhanced tau clearance approach in synapses via the synaptic proteasome system in PS19 mice. We had previously shown that activating a peptidergic Gs-GPCR, the PAC1 receptor (PAC1R) present post-synaptically could stimulate cAMP/PKA-mediated proteasome activity and enhance the turnover of toxic synaptic tau and overall tauopathy (Schaler et al., 2021). However, peptide-related therapies have limited clinical application due to rapid enzymatic degradation and diminished concentration in the brain. To overcome these limitations, in the present study, we targeted the 5-HT4 serotonergic receptor, a successful druggable target that is a promising target for preventing MCI in AD patients (de Cates et al., 2021). Targeting the serotonin innervation to strengthen synapses has been recognized as an early defense against memory decline and loss of the serotonin system in hippocampal and cortical regions, which is evident in AD (Smith et al., 2017). Of all the 5-HT receptors (5-HTRs) subfamilies, 5-HT4R has been studied extensively in preclinical studies as a pro-cognitive target (Lamirault and Simon, 2001; Darcet et al., 2016). At the behavioral level, administration of 5-HT4R agonists improves or restores hippocampal-dependent memory of aged and amyloid-producing AD models (Baranger et al., 2017). At the cellular and molecular levels, 5-HT4R activation with small molecule agonists leads to synaptogenic, neurotrophic, and neuroprotective effects through well-documented mechanisms of production of non-amyloidogenic sAPPα, activation of cAMP/PKA/CREB signaling, and enhanced BDNF expression (Lucas et al., 2007; Restivo et al., 2008; Cochet et al., 2013; Hagena and Manahan-Vaughan, 2017). The most recent FDA-approved 5-HT4R partial agonist, prucalopride, has been tested short term in 44 healthy human volunteers showing improved episodic memory and increased hippocampus circuitry during memory retrieval compared to placebo (de Cates et al., 2021).

While the molecular signature of 5-HT4R stimulation in the CNS has been reported through the anti-amyloidogenic cascade (Cochet et al., 2013) and the synapse to nucleus pro-cognitive and neurotrophic effect (Hagena and Manahan-Vaughan, 2017), there are no data reported on the impact of 5-HT4R activation on tau pathology.

Here, we tested the long-term administration of the 5-HT4R partial agonists, prucalopride, and RS-67333 on the PS19 tauopathy mouse model. Our results expand the therapeutic potential of the 5-HT4R by showing that 5-HT4R agonists treatment could reduce tau burden and overall tauopathy in the brain. Furthermore, since 5-HT4R is present predominately post-synaptically, we hypothesized that its agonism could enhance the signaling cascade to mediate enhanced proteasome activity and reduce tau restricted to local stimulation in synapses. Indeed, our results show a significant reduction in synaptic tau in the agonist treatment conditions. Over time, enhanced synaptic tau turnover can lead to reduced tauopathy throughout the brain. Consistent with our previous studies demonstrating cAMP/PKA mediated activation of proteasome function (Myeku et al., 2016; Schaler et al., 2021), here we show that administration of 5-HT4R agonists leads to increased activity of the proteasome, resulting in reduced ubiquitinated proteins in synapses. Moreover, the behavioral testing showed that 5-HT4R agonism had an anxiolytic effect on female PS19 mice. Sex differences in 8–12-month-old PS19 mice have been reported previously (Sun et al., 2020). Importantly we detected improved hippocampal-related behavioral tasks on both sexes on agonists treated PS19 group, providing further support for a pro-cognitive effect of 5-HT4R agonism. The importance of 5-HT4R-directed synaptic therapies was emphasized by our bioinformatics results showing that the 5-HT4R serotonergic pathway is downregulated predominantly in neurons, reflecting the occurrence of serotonergic degeneration in AD brain (Smith et al., 2017). Thus, preservation of 5-HT4R and its signaling cascade by agonists starting from the asymptotic stages of the disease could delay cognitive decline and tau aggregation in AD.

There are several limitations to our study. The behavioral tests used in PS19 mice may not capture the full range of cognitive and neurological changes associated with tauopathy. Additionally, the relevance of these tests to human cognitive functions in AD may have limitations. Cognitive enhancement by the PKA/CREB pathway may also be a confounding factor, and improved cognition may not solely reflect the enhanced proteasome degradation and reduced tauopathy. Moreover, the study shows behavioral differences between male and female PS19 mice, particularly in response to 5-HT4R agonist treatment. These sex-dependent effects suggest that the efficacy and therapeutic mechanisms of 5-HT4R agonists might vary between males and females, necessitating further investigation.

While the study highlights the role of 5-HT4R stimulation in reducing tau pathology and enhancing proteasome activity, the precise molecular mechanisms underlying these effects could be explored further to understand the therapeutic potential and limitations of 5-HT4R agonism fully.

Like most drug candidates, 5-HT4 agonists come with the potential for adverse effects. The safety profile varies among 5-HT4 agonists, exhibiting various cardiac and gastrointestinal safety profiles. 5-HT4 agonists vary among different compounds, with some showing minimal cardiac effects and others being associated with more significant concerns. However, being the latest 5-HT4 agonist in the market, prucalopride has a favorable safety profile, particularly cardiovascular safety, distinguishing it from older serotonin agonists.

## Conclusion

Altogether, our results show the therapeutic potential of targeting 5-HT4R in preserving synapse-to-nucleus serotonergic pathways, which are downregulated in AD. We show that maintaining synapse integrity by reducing the toxic accumulation of synaptic tau and enhancing plasticity-related memory could be crucial to identifying viable targets for early AD treatments.

## Data availability statement

Bulk-seq data from MSBB (https://www.synapse.org/#!Synapse:syn20801188); bulk-seq data from ROSMAP (https://www.synapse.org/#!Synapse:syn23650893); bulk-seq data from Mayo (https://www.synapse.org/#!Synapse:syn5550404); snRNA-seq data from Mathys et al. (2019) (https://www.synapse.org/#!Synapse:syn18485175); and snRNA-seq data from SEA-AD (https://cellxgene.cziscience.com/collections/1ca90a2d-2943-483d-b678-b809bf464c30).

## Ethics statement

All animal procedures of experiments were done in accordance with guidelines from the NIH animal use and care policies and with the approval of the Columbia University IACUC. The study was conducted in accordance with the local legislation and institutional requirements.

## Author contributions

NM: Conceptualization, Formal analysis, Funding acquisition, Investigation, Resources, Supervision, Writing – original draft, Writing – review & editing. SJ: Data curation, Methodology, Software, Writing – review & editing. ES: Methodology, Software, Writing – review & editing. AR: Data curation, Methodology, Writing – review & editing. RS: Data curation, Methodology, Writing – review & editing. MS: Data curation, Methodology, Writing – review and editing. HF: Methodology, Writing – review & editing. MY: Data curation, Formal analysis, Investigation, Methodology, Writing – review & editing.

## Footnotes

1
http://rsb.info.nih.gov/ij

2
https://adknowledgeportal.synapse.org/

3
https://cellxgene.cziscience.com/collections/
