# Correction: 5-HT4 receptor agonists treatment reduces tau pathology and behavioral deficit in the PS19 mouse model of tauopathy

**DOI:** 10.3389/fncel.2026.1945844

**Published:** 2026-08-11

**Authors:** Shan Jiang, Eric J. Sydney, Avery M. Runyan, Rossana Serpe, Malavika Srikanth, Helen Y. Figueroa, Mu Yang, Natura Myeku

**Affiliations:** 1 Taub Institute for Research on Alzheimer's Disease and the Aging Brain, Columbia University Irving Medical Center, New York, NY, United States; 2 Department of Pathology and Cell Biology, Columbia University Irving Medical Center, New York, NY, United States; 3 The Institute for Genomic Medicine and Psychiatry, Columbia University Irving Medical Center, New York, NY, United States

**Keywords:** Alzheimer's disease, ubiquitin proteasome system (UPS), tauopathy, 5-HT4 agonist, synaptic therapy

There was a mistake in Figure 5E as published. In the published article, the *p*-value annotation boxes in Figure 5E were inadvertently displaced vertically from their intended positions during final figure assembly. This affected only the placement of the *p*-value annotations and did not alter the underlying circular heatmap, statistical values, analyses, or scientific conclusions. The corrected Figure 5E appears below.

The original version of this article has been updated.

**FIGURE 5:**
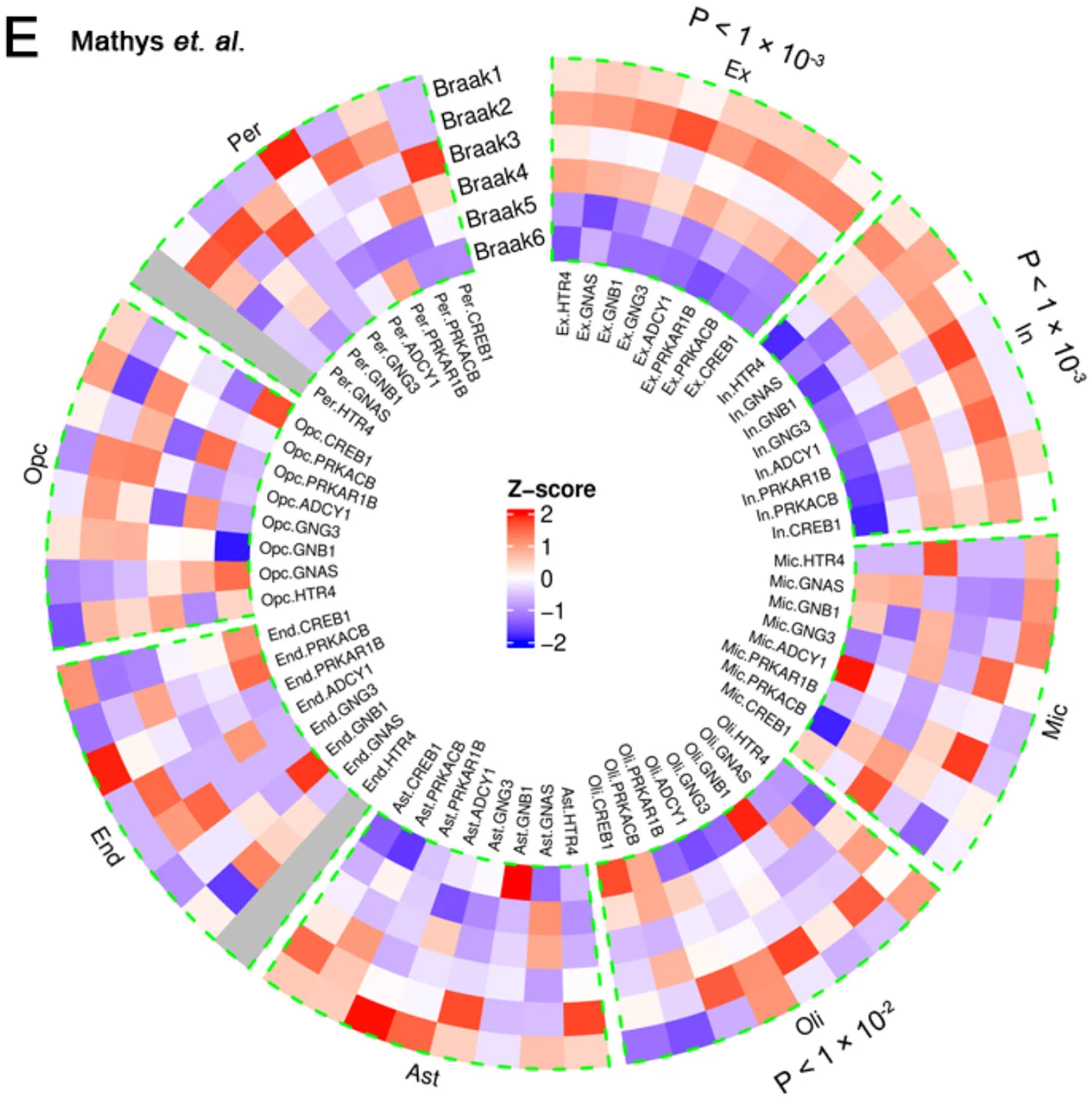
The RNA-seq analyses show decreased expression of 5-HT4R/AC/PKA/CREB1 pathway-related genes with exacerbated tau aggregation. (A–C) Bulk RNA-seq data analyses presented as heatmaps showing HTR4/AC/PKA/CREB1 pathway-related genes (except CREB1) were decreased in advanced Braak stages in the AD post-mortem brains. Brain areas (A) parahippocampal gyrus (PHG) from MSBB study. (B) Dorsolateral prefrontal cortex from the ROSMAP study. (C) Temporal cortex from the Mayo study. Gene expressions were scaled with Z-score transformation. Ordinal regression was used to assess the correlation between Braak stages and gene expression after adjusting for the effects of confounding factors such as age, gender, ethnicity, PMI, RIN, and library size. *P < 0.05, **P < 0.01, and ***P < 0.001 after FDR correction. (D) Scatter plot showing the HTR4 expression was negatively correlated with Ab plaque measure in PHG from MSBB study with linear regression after adjusting for confounding effects. (E, F) Single nucleus RNA-seq analyses presented in circular heatmaps show the expression percent of HTR4/AC/PKA/CREB1 pathway-related genes were decreased with advancing Braak stages in excitatory and inhibitory neurons. (E) Prefrontal cortex from Mathys et al. (2019) dataset study. (F) The middle temporal gyrus from SEA-AD studies and Gene expression percentages were scaled with Z-score transformation. SnRNA-seq data were analyzed using the Kruskal–Wallis test; *P < 0.05, **P < 0.01, and ***P < 0.001 after FDR correction. Ex, excitatory neurons; In, inhibitory neurons; Mic, microglia; Oli, oligodendrocytes; Ast, astrocytes; End, endothelial cells; Opc, oligodendrocyte progenitor cells; Per, pericytes; VLMC, vascular leptomeningeal cells.

