# Supplementary material for: 5-HT4 receptor agonists treatment reduces tau pathology and behavioral deficit in the PS19 mouse model of tauopathy
Source: Front Cell Neurosci. 2024 Apr 4;18:1338502. doi: 10.3389/fncel.2024.1338502 (PMC11024353; doi:10.3389/fncel.2024.1338502)
Supplement: Supplementary file 1 [file Data_Sheet_1.PDF]

Supplementary Materials

5-HT4 receptor agonists treatment reduces tau pathology and behavioral deficit in the PS19 mouse model of tauopathy

Shan Jiang[1,2], Eric J. Sydney[1,2], Avery M. Runyan[1,2], Rossana Serpe[1,2], Malavika Srikanth [1,2], Helen Y. Figueroa[1,2] , Mu Yang [3], Natura Myeku[1,2*]

[1] Taub Institute for Research on Alzheimer's Disease and the Aging Brain, Columbia University Irving Medical Center, New York, NY 10032, USA.
[2] Department of Pathology and Cell Biology, Columbia University Irving Medical Center, New York, NY 10032, USA.
[3] The Institute for Genomic Medicine and Psychiatry, Columbia University Irving Medical Center, New York, NY 10032, USA.
*Corresponding author. Email: nm2631@cumc.columbia.edu

Fig. S1. 5-HT4 receptor agonists treatment attenuates tauopathy- Study 1.
Fig. S2. 5-HT4 receptor agonists treatment attenuates tauopathy- Study 2.
Fig. S3. 5-HT4 stimulation reduced tau in young wild-type mice.
Fig. S4. Meta-analysis by forest plots combining the three bulk RNA-seq datasets.

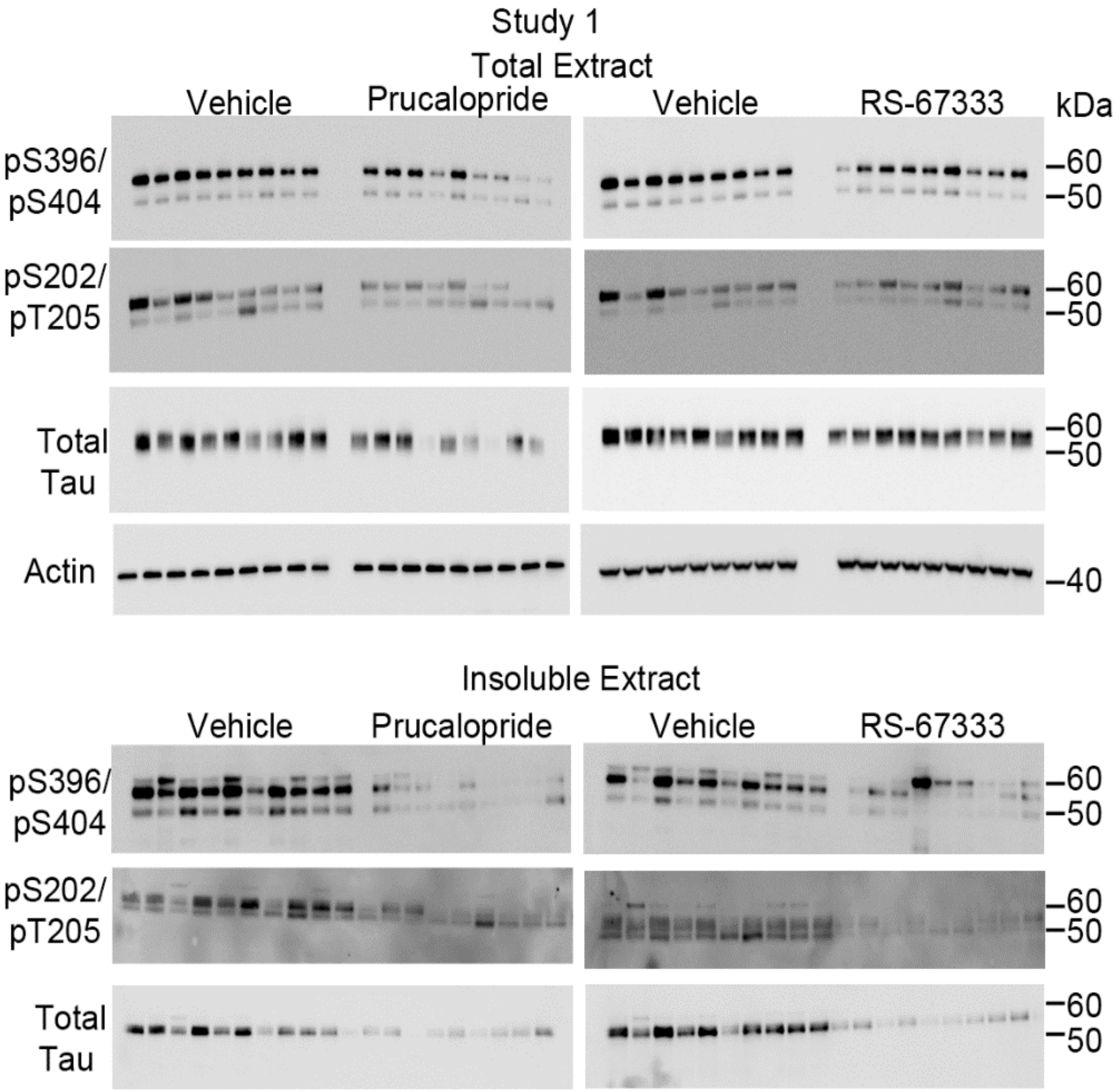

Supplementary Figure 1

Fig. S1. 5-HT4 receptor agonists treatment attenuates tauopathy- Study 1.
Summary of immunoblotting results from Fig.1. In vivo treatments were carried out in two sets/studies.

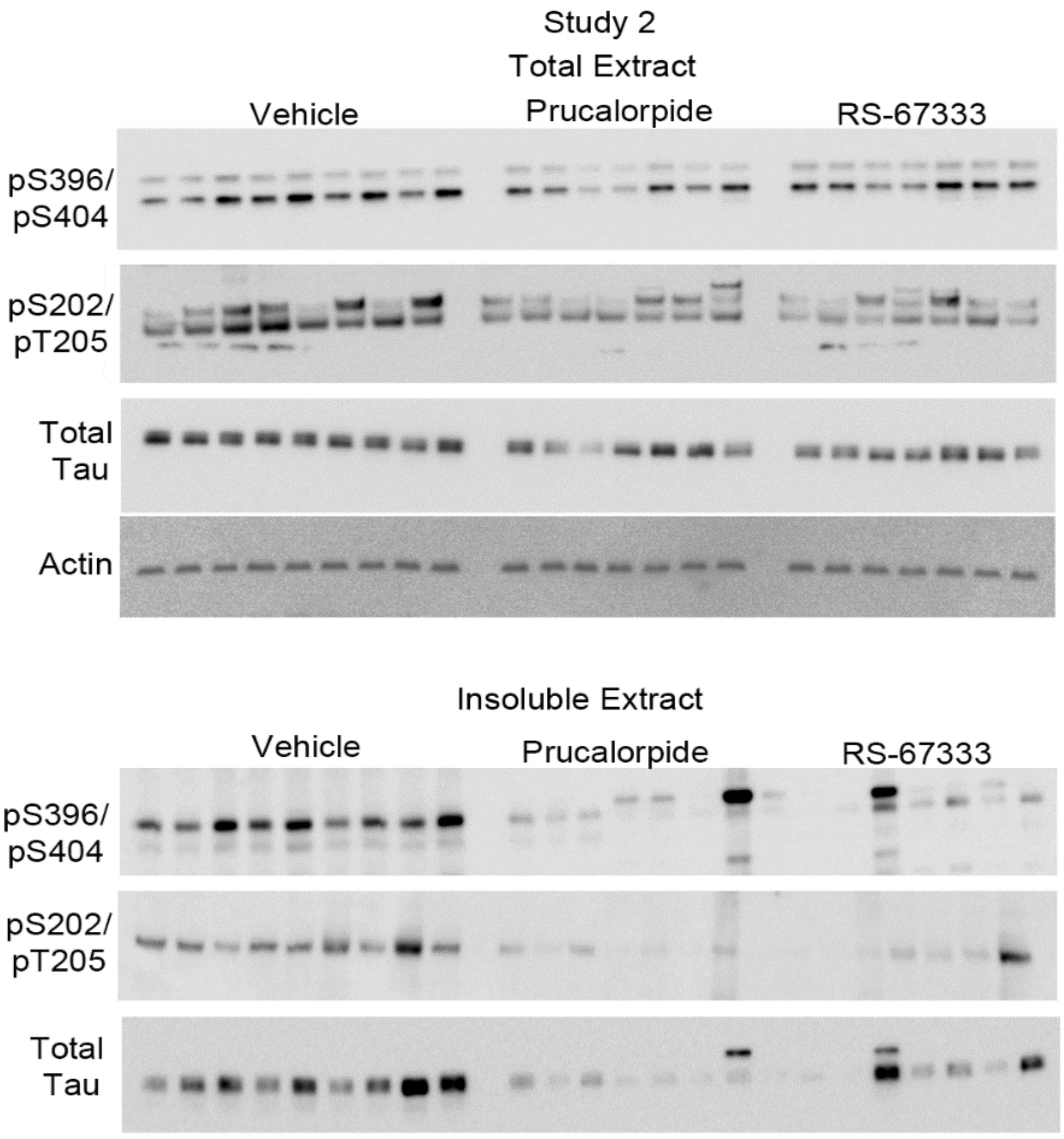

Supplementary Figure 2

Fig. S2. 5-HT4 receptor agonists treatment attenuates tauopathy- Study 2.
Summary of immunoblotting results from Fig.1. In vivo treatments were carried out in two sets/studies.

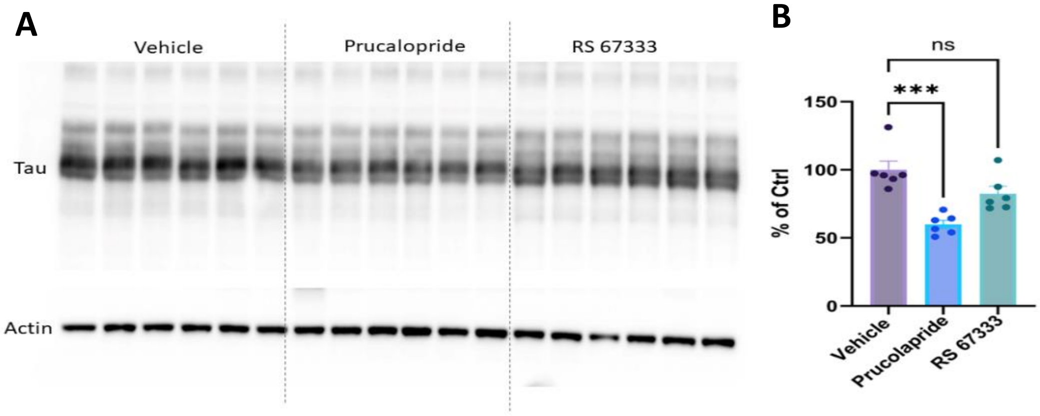

Supplementary Figure 3

Fig. S3. 5-HT4 stimulation reduced tau in young wild-type mice.
6-7 months old WT mice treated with vehicle, prucalopride, and RS 67333. A) Western blotting for murine tau and actin as a control. B) Quantification of western blotting for A. N=6 for each treated group.

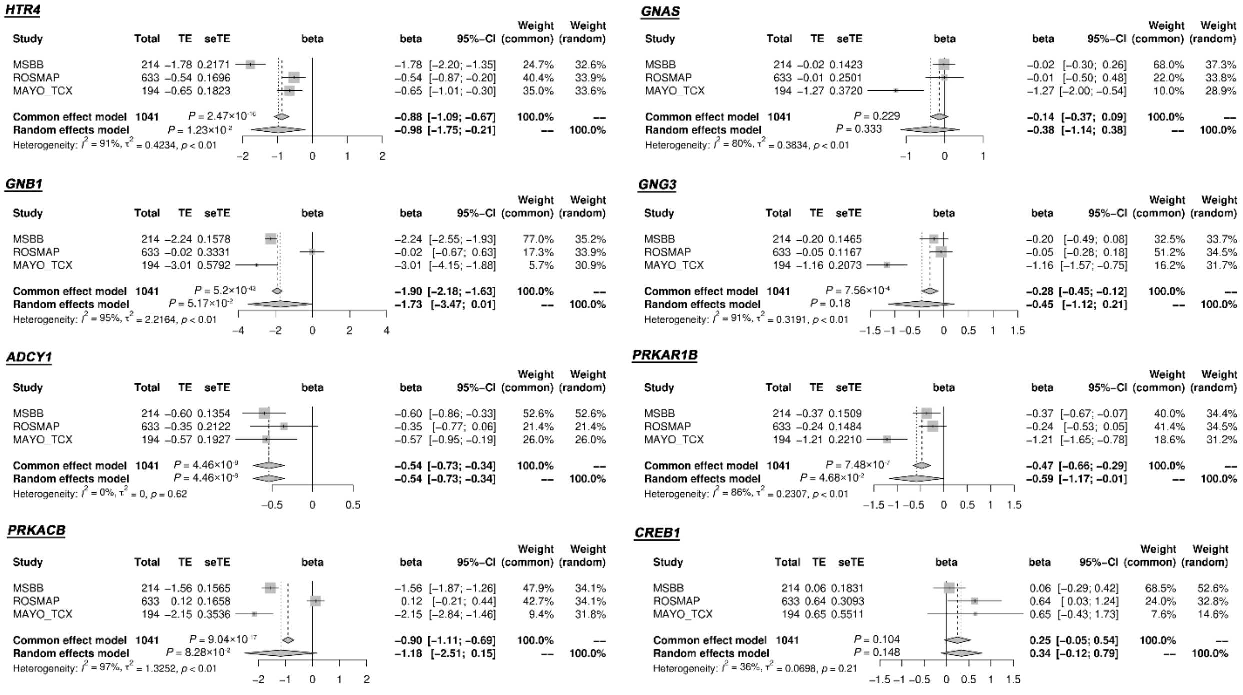

Supplementray Figure 3

Fig. S4. Meta-analysis by forest plots combining the three bulk RNA-seq datasets.
Data are point estimates of beta coefficients and a range of two-sided 95% confidence intervals (CI) of the beta coefficients. Heterogeneity among datasets was assessed with I^2 index. Combined beta coefficients and the corresponding P values were calculated in both fixed and random effect models.
